# Bibliometric analysis of the top-50 cited articles on COVID-19 and physical activity

**DOI:** 10.3389/fpubh.2022.926244

**Published:** 2022-09-09

**Authors:** Fan Zhang, Ying Zhang, Yaqi Yu, Wei Lu, Huachun Zhang

**Affiliations:** ^1^Department of Nephrology, Longhua Hospital Shanghai University of Traditional Chinese Medicine, Shanghai, China; ^2^Department of Surgery, Longhua Hospital Shanghai University of Traditional Chinese Medicine, Shanghai, China; ^3^Department of Cancer, Longhua Hospital Shanghai University of Traditional Chinese Medicine, Shanghai, China; ^4^Department of Nursing, Longhua Hospital Shanghai University of Traditional Chinese Medicine, Shanghai, China

**Keywords:** COVID-19, physical activity, bibliometric analysis, citation classics, top-50

## Abstract

**Background:**

Since the 2019 novel coronavirus (COVID-19) pneumonia outbreak in late 2019, an endless stream of research has emerged surrounding physical activity. This study analyzes the 50 most influential articles on COVID-19 and physical activity over the past 2 years to describe the research landscape and hotspots from bibliometric citation analysis.

**Methods:**

The top-50 cited articles were extracted from the Web of Science Core Collection database, and bibliometric citation analysis was performed by Excel 2019 and VOSviewer software.

**Results:**

The top-50 articles were cited 160.48 ± 106.90 (range: 70–587). Most of the articles were from the United States (14), followed by Italy (11) and England (9). The *International Journal of Environmental Research and Public Health* (*n* = 10) is the journal with the top-50 cited articles. The collaboration between authors was mainly among three teams, including Smith L, Musumeci G, and Napoli C. The hotspot of research around COVID-19 and physical activity focused on lifestyle change (sedentary behavior, sitting time), mental health (depressive, anxiety, loneliness), the credibility of physical activity assessment tools (reliability, validity), and physical activity of different populations (gender, youth, children).

**Conclusions:**

Based on a bibliometric analysis of high-impact articles on COVID-19 and physical activity highlights physical activity as an essential lifestyle change and developments and hotspots in this field. These data will provide insights for future researchers regarding the direction of physical activity research in the COVID-19 pandemic.

## Introduction

2019 novel coronavirus (COVID-19), first reported in December 2019 in Wuhan, China, by a patient with pneumonia (1), has surpassed Middle East respiratory syndrome coronavirus and severe acute respiratory syndrome coronavirus in terms of transmission in the population (2). As of April 10, 2022, nearly 500 million confirmed cases of COVID-19, including 6.2 million deaths, have been reported in China and at least 85 other countries and/or regions (3). The human health risks of COVID-19 include direct harm to the respiratory system, damage to the immune system, worsening of the underlying disease, and ultimately systemic failure and death (4–6).

The COVID-19 outbreak turned the life of people around the world upside down (7–9). During the COVID-19 pandemic, not only were large numbers of patients hospitalized, but tens of thousands of people were forced into isolation in limited spaces (10). This dramatic change in lifestyle caused by immobilization (hospitalization and bed rest), isolation, and lack of physical activity could result in the second wave of attacks on the health and wellbeing of both infected and general populations (11–14). In this context, scholars from various countries have conducted a series of studies on COVID-19 and physical activity, addressing the role and impact of physical activity during the COVID-19 pandemic from different perspectives (15–17).

A citation is an article (citation) that cites another article (cited citation) as a reference. The number of citations is not only a measure of an article's impact on the scientific community but is also the basis for generating a journal's impact factor (IF) (18). Eugene Garfield introduced the term “citation classics” in 1955 to identify the most cited scientific articles in the Institute for Scientific Information (ISI) Web of Knowledge (now known as Web of Science) database (19). In most fields, an article cited more than 100 times is considered a citation classic (19). Reviewing the most cited articles (the so-called “citation classics”) can provide interesting information about scientific progress and research trends in a particular subject area (20).

Given that the publication of COVID-19 and physical activity is rapidly evolving, its scientific dynamics and profile deserve to be observed. Therefore, the primary objective of this review is to identify the 50 most-cited articles in the field of COVID-19 with physical activity based on a forward citation analysis, and the secondary objective is to visualize countries, authors, and keywords through VOSviewer software to increase the understanding of the current status of research and hot spots to inform the study priorities in the field.

## Materials and methods

### Search strategy

We selected the Web of Science Core Collection database as the publication source. The database encompasses more than 20,000 peer-reviewed, high-quality scholarly journals, including open access journals published in more than 250 medical, social science, and humanities disciplines worldwide, and is widely used for bibliometric analysis. In addition, the database provides the authors, countries, and keywords for each publication, which was necessary for this study (21). Since COVID-19 broke out in December 2019, our search dates are set for January 1, 2020, to April 9, 2022.

The search formula was TS = (“COVID-19” OR “SARS-CoV-2” OR “2019- nCoV” OR “2019 coronavirus” OR “2019 novel coronavirus”) AND TS = (“physical activity” OR “sedentary behaviors” OR “sedentary lifestyle” OR “physical inactivity” OR “sedentary time” OR “step per day” OR “steps per day” OR “step count” OR “step/day” OR “steps/day” OR “step/d” OR “steps/d” OR “daily step” OR “daily steps” OR “accelerometer” OR “pedometer”).

### Publication selection and data extraction

**Figure 1:**
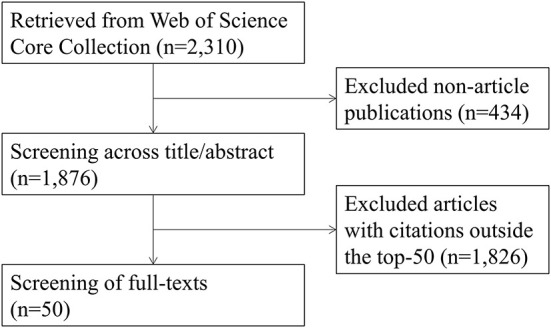
Flow chart of literature screening.

First, the document type was restricted to “article” from the 2,310 publications initially retrieved, and then the title and abstract of each article were independently reviewed by two authors to ensure they were relevant to COVID-19 and physical activity. Then the top-50 cited articles were selected for bibliometric analysis based on the citation number sorting with 50 as the cut-off value. Our study had no restrictions on study population, design, or language. When non-English publications were encountered, we used Deepl for translation. The flow chart of the study is shown in Figure 1.

The 50 most cited articles were reviewed, and the following information was extracted: (1) author; (2) year of publication; (3) journal; (4) impact factor (2021); (5) total citations; (6) annual citations.

### Visualization

All information and data for each article are inserted into Microsoft Excel 2019 and VOSviewer (version 1.6.15). VOSviewer is a Java-based bibliometric networks analysis software, mainly for literature data, applicable to unimodal undirected network, focusing on the visualization of scientific knowledge. Co-authorship analysis is used to depict author and country collaboration. Co-occurrence analysis of keywords is performed to detect research hotspots.

### Statistical analysis

Data are presented by using descriptive statistic and no statistical significance tests were performed.

## Result

The 50 most cited articles in COVID-19 and physical activity research and the number of citations are shown in Table 1. The number of citations ranged from 70 to 587 (mean 160.48 ± 106.90; median: 131) and the annual citations ranged from 24.33 to 195.67 (mean 55.34 ± 35.48; median: 45.84).

**Table 1:** List of the top-50 cited articles.

**Author (year)**	**Title**	**Journal**	**Total citations**	**Average per year**	**References**
Ammar et al. (22)	Effects of COVID-19 Home Confinement on Eating Behavior and Physical Activity: Results of the ECLB-COVID19 International Online Survey	Nutrients	587	195.67	(22)
Di Renzo et al. (23)	Eating habits and lifestyle changes during COVID-19 lockdown: an Italian survey	Journal of Translational Medicine	471	157	(23)
Stanton et al. (24)	Depression, Anxiety and Stress during COVID-19: Associations with Changes in Physical Activity, Sleep, Tobacco and Alcohol Use in Australian Adults	International Journal of Environmental Research and Public Health	423	141	(24)
Shechter et al. (25)	Psychological distress, coping behaviors, and preferences for support among New York healthcare workers during the COVID-19 pandemic	General Hospital Psychiatry	339	113	(25)
Moore et al. (26)	Impact of the COVID-19 virus outbreak on movement and play behaviors of Canadian children and youth: a national survey	International Journal of Behavioral Nutrition and Physical Activity	294	98	(26)
Pieh et al. (27)	The effect of age, gender, income, work, and physical activity on mental health during coronavirus disease (COVID-19) lockdown in Austria	Journal of Psychosomatic Research	291	97	(27)
Maugeri et al. (28)	The impact of physical activity on psychological health during COVID-19 pandemic in Italy	Heliyon	263	87.67	(28)
Lesser and Nienhuis (29)	The Impact of COVID-19 on Physical Activity Behavior and Well-Being of Canadians	International Journal of Environmental Research and Public Health	223	74.33	(29)
Dunton et al. (30)	Early effects of the COVID-19 pandemic on physical activity and sedentary behavior in children living in the US	BMC Public Health	213	71	(30)
Lebel et al. (31)	Elevated depression and anxiety symptoms among pregnant individuals during the COVID-19 pandemic	Journal of Affective Disorders	205	68.33	(31)
Huckins et al. (32)	Mental Health and Behavior of College Students During the Early Phases of the COVID-19 Pandemic: Longitudinal Smartphone and Ecological Momentary Assessment Study	Journal of Medical Internet Research	196	65.33	(32)
Nguyen et al. (33)	People with Suspected COVID-19 Symptoms Were More Likely Depressed and Had Lower Health-Related Quality of Life: The Potential Benefit of Health Literacy	Journal of Clinical Medicine	194	64.67	(33)
Hamer et al. (34)	Lifestyle risk factors, inflammatory mechanisms, and COVID-19 hospitalization: A community-based cohort study of 387,109 adults in UK	Brain Behavior and Immunity	184	61.33	(34)
Meyer et al. (35)	Changes in Physical Activity and Sedentary Behavior in Response to COVID-19 and Their Associations with Mental Health in 3052 US Adults	International Journal of Environmental Research and Public Health	172	57.33	(35)
Robinson et al. (36)	Obesity, eating behavior and physical activity during COVID-19 lockdown: A study of UK adults	Appetite	171	85.5	(36)
Zachary et al. (37)	Self-quarantine and weight gain related risk factors during the COVID-19 pandemic	Obesity Research & Clinical Practice	161	53.67	(37)
Mattioli et al. (38)	Quarantine during COVID-19 outbreak: Changes in diet and physical activity increase the risk of cardiovascular disease	Nutrition Metabolism and Cardiovascular Diseases	158	52.67	(38)
Newby et al. (39)	Acute Mental Health Responses During the COVID-19 Pandemic in Australia	PLoS One	153	51	(39)
Narici et al. (40)	Impact of sedentarism due to the COVID-19 home confinement on neuromuscular, cardiovascular and metabolic health: Physiological and pathophysiological implications and recommendations for physical and nutritional countermeasures	European Journal of Sport Science	148	49.33	(40)
Zhang et al. (41)	Mental Health Problems during the COVID-19 Pandemics and the Mitigation Effects of Exercise: A Longitudinal Study of College Students in China	International Journal of Environmental Research and Public Health	145	48.33	(41)
Sepulveda-Loyola et al. (42)	Impact of Social Isolation Due to COVID-19 on Health in Older People: Mental and Physical Effects and Recommendations	Journal of Nutrition Health & Aging	143	47.67	(42)
Carroll et al. (43)	The Impact of COVID-19 on Health Behavior, Stress, Financial and Food Security among Middle to High Income Canadian Families with Young Children	Nutrients	140	46.67	(43)
Constandt et al. (44)	Exercising in Times of Lockdown: An Analysis of the Impact of COVID-19 on Levels and Patterns of Exercise among Adults in Belgium	International Journal of Environmental Research and Public Health	138	46	(44)
Barker-Davies et al. (45)	The Stanford Hall consensus statement for post-COVID-19 rehabilitation	British Journal of Sports Medicine	137	45.67	(45)
Gornicka et al. (46)	Dietary and Lifestyle Changes During COVID-19 and the Subsequent Lockdowns among Polish Adults: A Cross-Sectional Online Survey PLifeCOVID-19 Study	Nutrients	131	43.67	(46)
Pecanha et al. (47)	Social isolation during the COVID-19 pandemic can increase physical inactivity and the global burden of cardiovascular disease	American Journal of Physiology-Heart and Circulatory Physiology	131	43.67	(47)
Deschasaux-Tanguy et al. (48)	Diet and physical activity during the coronavirus disease 2019 (COVID-19) lockdown (March-May 2020): results from the French NutriNet-Sante cohort study	American Journal of Clinical Nutrition	127	63.5	(48)
Castaneda-Babarro et al. (49)	Physical Activity Change during COVID-19 Confinement	International Journal of Environmental Research and Public Health	125	41.67	(49)
Mattioli et al. (50)	COVID-19 pandemic: the effects of quarantine on cardiovascular risk	European Journal of Clinical Nutrition	125	41.67	(50)
Sallis et al. (51)	Physical inactivity is associated with a higher risk for severe COVID-19 outcomes: a study in 48 440 adult patients	British Journal of Sports Medicine	115	57.5	(51)
Sanchis-Gomar et al. (52)	Obesity and Outcomes in COVID-19: When an Epidemic and Pandemic Collide	Mayo Clinic Proceedings	114	38	(52)
Majumdar et al. (53)	COVID-19 pandemic and lockdown: cause of sleep disruption, depression, somatic pain, and increased screen exposure of office workers and students of India	Chronobiology International	105	35	(53)
Flanagan et al. (54)	The Impact of COVID-19 Stay-At-Home Orders on Health Behaviors in Adults	Obesity	101	33.67	(54)
Schmidt et al. (55)	Physical activity and screen time of children and adolescents before and during the COVID-19 lockdown in Germany: a natural experiment	Scientific Reports	101	33.67	(55)
Cheval et al. (56)	Relationships between changes in self-reported physical activity, sedentary behavior and health during the coronavirus (COVID-19) pandemic in France and Switzerland	Journal of Sports Sciences	99	33	(56)
Galle et al. (57)	Understanding Knowledge and Behaviors Related to COVID-19 Epidemic in Italian Undergraduate Students: The EPICO Study	International Journal of Environmental Research and Public Health	92	30.67	(57)
Yamada et al. (58)	Effect of the COVID-19 Epidemic on Physical Activity in Community-Dwelling Older Adults in Japan: A Cross-Sectional Online Survey	Journal of Nutrition Health & Aging	91	30.33	(58)
Bates et al. (59)	COVID-19 Impact on Behaviors across the 24-Hour Day in Children and Adolescents: Physical Activity, Sedentary Behavior, and Sleep	Children-Basel	90	30	(59)
Carter et al. (60)	Considerations for Obesity, Vitamin D, and Physical Activity Amid the COVID-19 Pandemic	Obesity	87	29	(60)
Lopez-Bueno et al. (61)	Health-Related Behaviors Among School-Aged Children and Adolescents During the Spanish COVID-19 Confinement	Frontiers in Pediatrics	86	28.67	(61)
Romero-Blanco et al. (62)	Physical Activity and Sedentary Lifestyle in University Students: Changes during Confinement Due to the COVID-19 Pandemic	International Journal of Environmental Research and Public Health	81	27	(62)
Van Rheenen et al. (63)	Mental health status of individuals with a mood-disorder during the COVID-19 pandemic in Australia: Initial results from the COLLATE project	Journal of Affective Disorders	80	26.67	(63)
Schuch et al. (64)	Associations of moderate to vigorous physical activity and sedentary behavior with depressive and anxiety symptoms in self-isolating people during the COVID-19 pandemic: A cross-sectional survey in Brazil	Psychiatry Research	79	26.33	(64)
Pillay et al. (65)	Nowhere to hide: The significant impact of coronavirus disease 2019 (COVID-19) measures on elite and semi-elite South African athletes	Journal of Science and Medicine in Sport	79	26.33	(65)
Gupta et al. (66)	Changes in sleep pattern and sleep quality during COVID-19 lockdown	Indian Journal of Psychiatry	75	25	(66)
Gallo et al. (67)	The Impact of Isolation Measures Due to COVID-19 on Energy Intake and Physical Activity Levels in Australian University Students	Nutrients	75	25	(67)
Galle et al. (68)	Sedentary Behaviors and Physical Activity of Italian Undergraduate Students during Lockdown at the Time of COVID-19 Pandemic	International Journal of Environmental Research and Public Health	73	24.33	(68)
Giustino et al. (69)	Physical Activity Levels and Related Energy Expenditure during COVID-19 Quarantine among the Sicilian Active Population: A Cross-Sectional Online Survey Study	Sustainability	73	24.33	(69)
Zheng et al. (70)	COVID-19 Pandemic Brings a Sedentary Lifestyle in Young Adults: A Cross-Sectional and Longitudinal Study	International Journal of Environmental Research and Public Health	70	35	(70)
Tornese et al. (71)	Glycemic Control in Type 1 Diabetes Mellitus During COVID-19 Quarantine and the Role of In-Home Physical Activity	Diabetes Technology & Therapeutics	70	35	(71)

**Figure 2:**
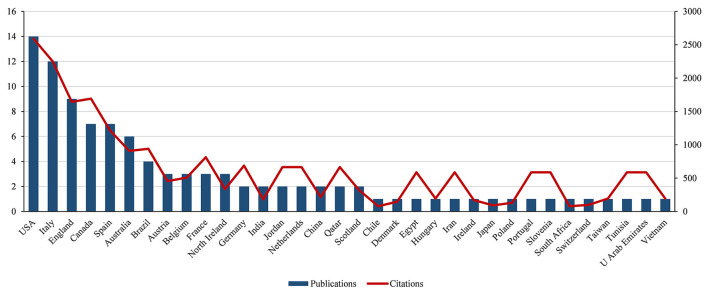
Number of top-cited articles by country/region.

**Figure 3:**
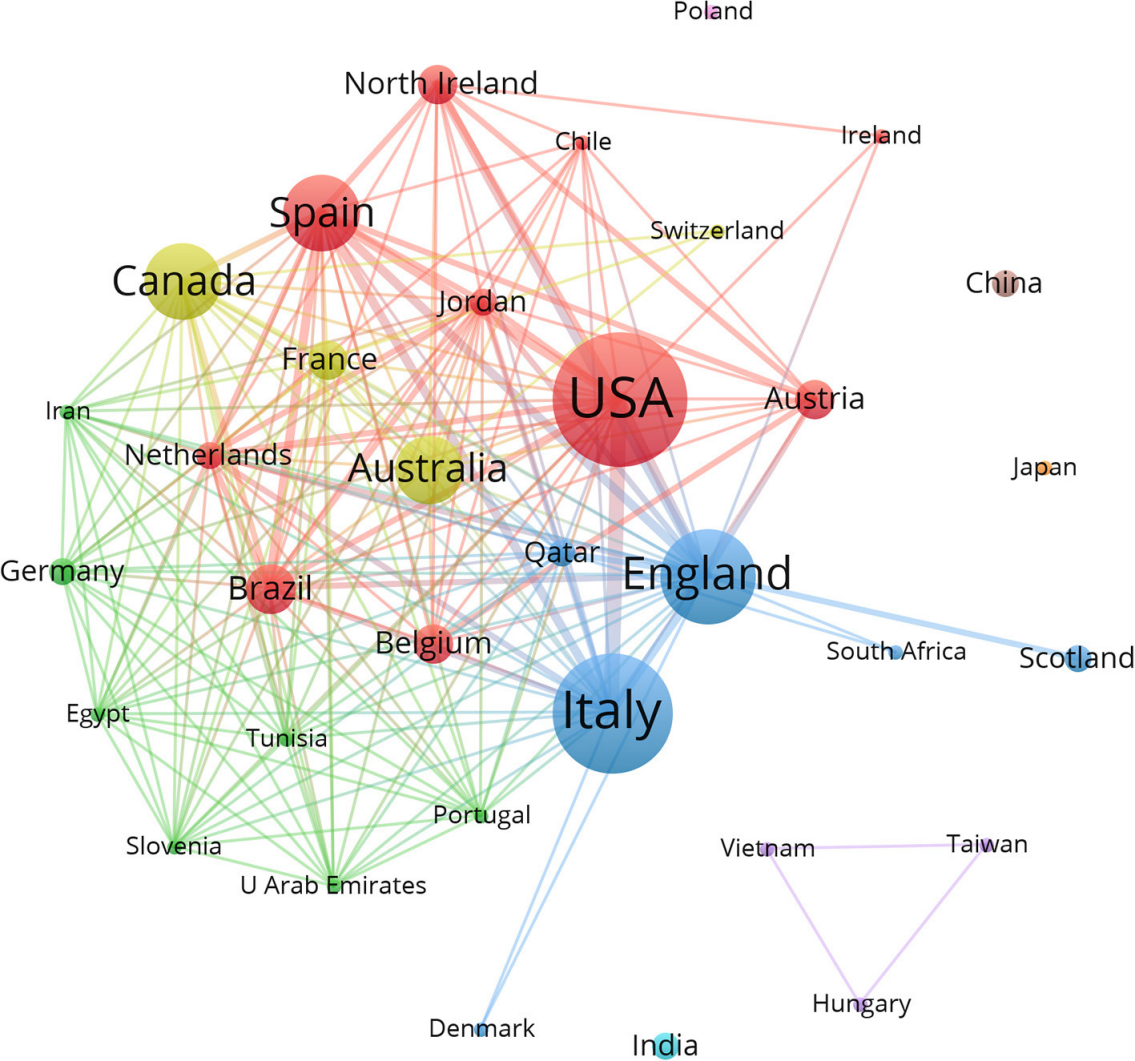
Countries/regions Cooperation Network Mapping.

The top-50 cited articles were published by authors from 34 countries (as shown in Figure 2) regarding country distribution. The United States contributed the highest number of articles (14), followed by Italy (11), England (9), Canada (8), and Spain (8). Figure 3 depicts the collaboration between the countries/regions that published the 50 most influential articles, showing close cooperation between a few countries and regions.

The 50 most cited articles were published in 34 journals with IF ranging from 1.759 to 13.800 (mean 4.55±2.17; median: 4.06) (Table 2). The *International Journal of Environmental Research and Public Health* (*n* = 10) is the journal with the most top-50 cited articles.

**Table 2:** Number of top-cited articles by journals.

**Journal**	**2020 IF**	**Publications**	**Citations**
International Journal of Environmental Research and Public Health	3.390	10	1,472
Nutrients	5.717	4	933
Journal of Nutrition Health & Aging	4.075	2	468
British Journal of Sports Medicine	13.800	2	252
Journal of Affective Disorders	4.839	2	285
Obesity	5.002	2	188
American Journal of Clinical Nutrition	7.045	1	127
American Journal of Physiology-Heart and Circulatory Physiology	4.733	1	131
Appetite	3.868	1	171
BMC Public Health	3.295	1	213
Brain Behavior and Immunity	7.217	1	184
Children-Basel	2.863	1	90
Chronobiology International	2.877	1	105
European Journal of Clinical Nutrition	4.016	1	125
European Journal of Sport Science	4.050	1	148
Frontiers in Pediatrics	3.418	1	86
General Hospital Psychiatry	3.238	1	339
Heliyon	2.749	1	263
Indian Journal of Psychiatry	1.759	1	75
International Journal of Behavioral Nutrition and Physical Activity	6.457	1	294
Journal of Clinical Medicine	4.241	1	194
Journal of Medical Internet Research	5.428	1	196
Journal of Psychosomatic Research	3.006	1	291
Journal of Science and Medicine in Sport	4.319	1	79
Journal of Sports Sciences	3.337	1	99
Journal of Translational Medicine	5.531	1	471
Mayo Clinic Proceedings	7.616	1	114
Nutrition Metabolism and Cardiovascular Diseases	4.222	1	158
Obesity Research & Clinical Practice	2.288	1	161
PLoS One	3.240	1	153
Psychiatry Research	3.222	1	79
Scientific Reports	4.379	1	101
Sustainability	3.251	1	73
Diabetes Technology & Therapeutics	6.118	1	70

**Figure 4:**
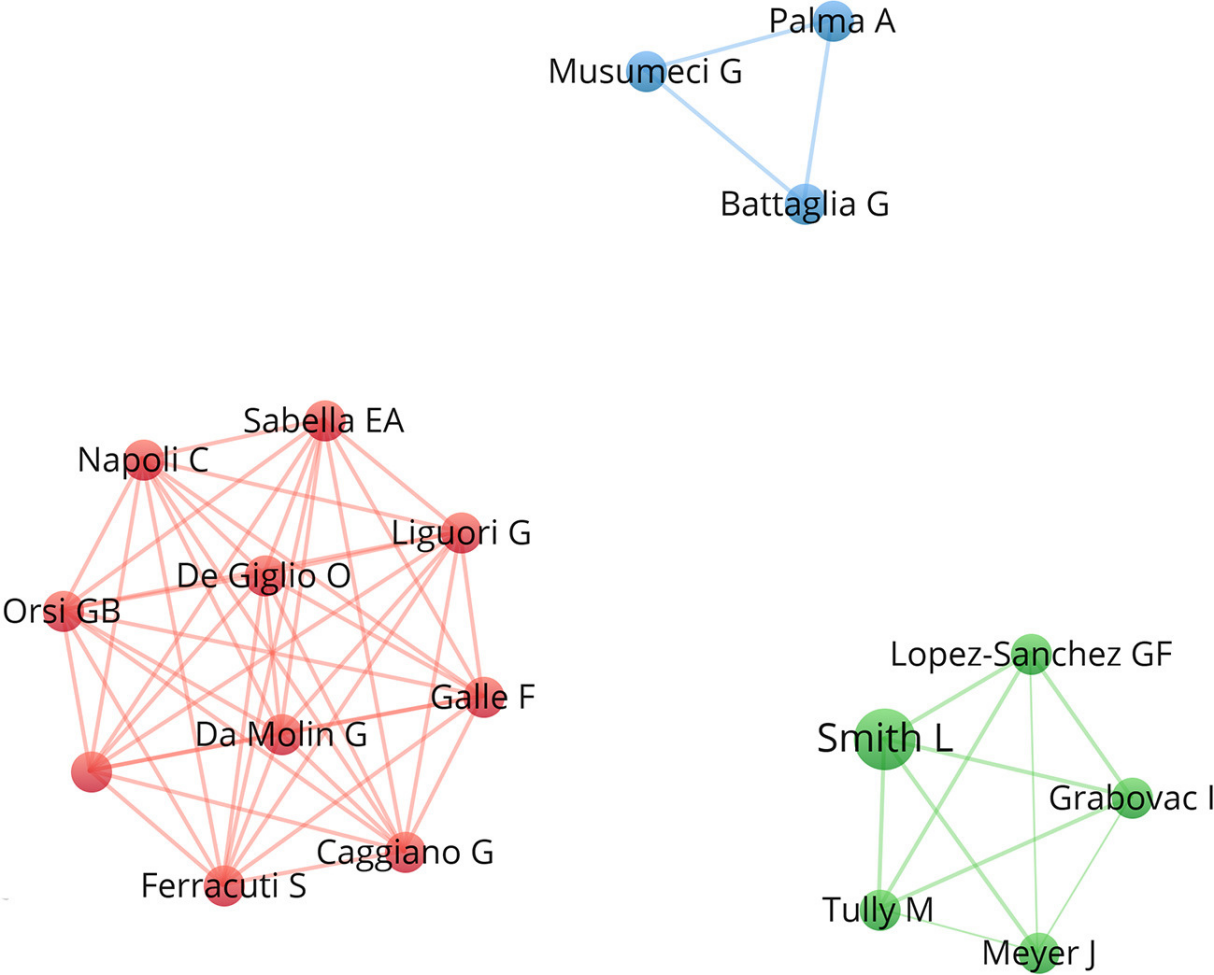
Authors Cooperation Network Mapping.

From the perspective of co-authorship, the collaboration network analysis divided authors into three clusters, identifying several major research teams, including Smith L, Musumeci G, and Napoli C (as shown in Figure 4).

**Figure 5:**
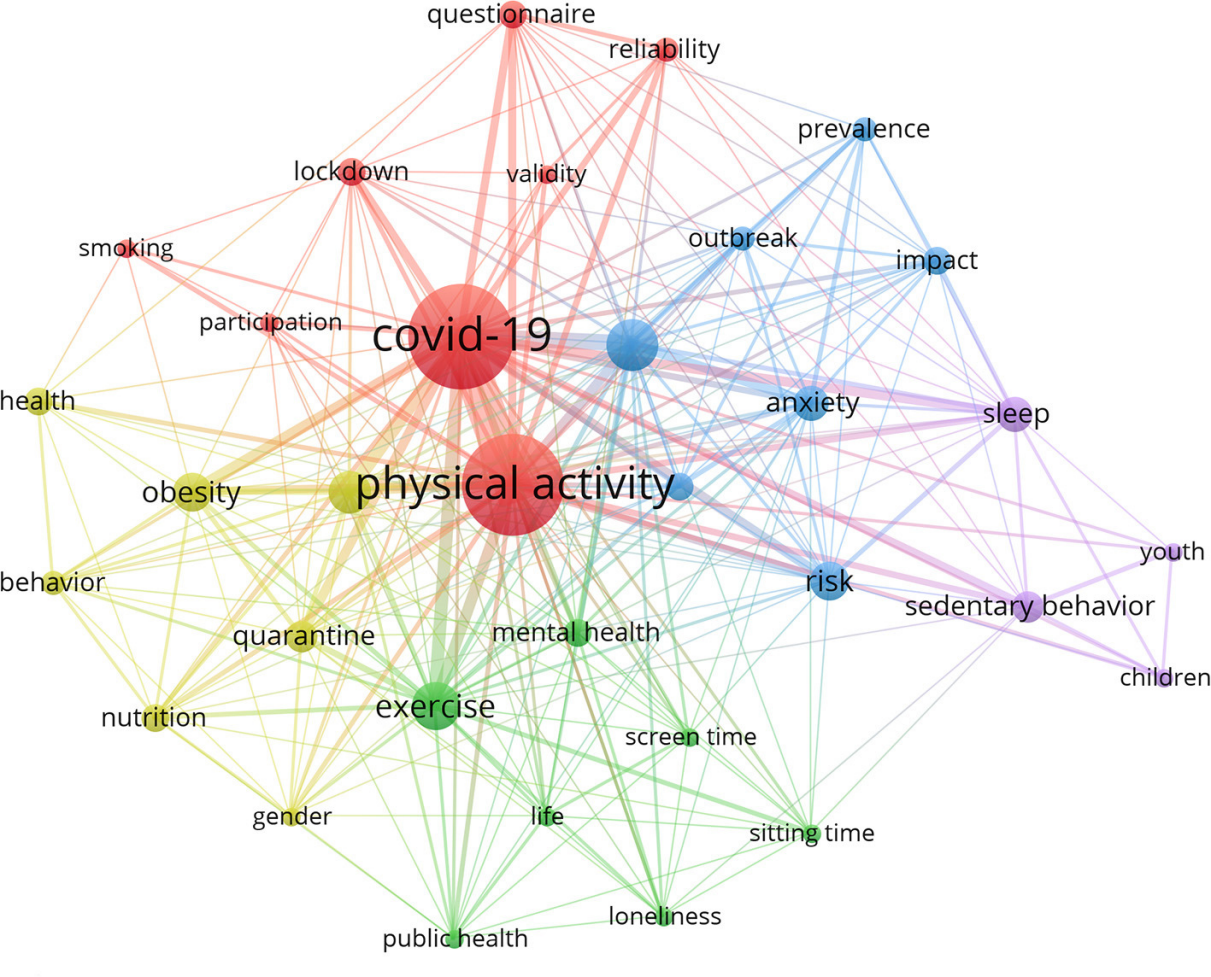
Keyword co-occurrence analysis.

Based on the keywords co-occurrence (Figure 5), the network mapping showed that the hotspots of research around COVID-19 and physical activity mainly focused on lifestyle change (sedentary behavior, sitting time), mental health (depressive, anxiety, loneliness), the credibility of physical activity assessment tools (reliability, validity), and physical activity of different populations (gender, youth, children).

## Discussion

Isolation at home during the prevalence of COVID-19 may prevent the virus from spreading but, in turn, brings a sedentary lifestyle (62). In terms of non-communicable disease prevention, physical activity is likely to be important for everyone, so it is more critical during outbreaks and blockades (72). Research around COVID-19 and physical activity has been gaining attention since the novel coronavirus outbreak in late 2019. This study describes the countries, journals, authors, and keywords of high-impact articles in this research area through citation analysis, showing the current trends.

The number of citations in this study was much lower than other physical activity-related citations, such as aging (73) and sleep (74) research. A major reason was the shorter duration. In addition, we extracted the average number of citations per year for different articles. This value can be used as a proxy for the total number of citations to assess the current impact of an article (75). The highest total citation was published in *Nutrients* in 2020 by Ammar et al. (22), which designed “Effects of home Confinement on multiple Lifestyle Behaviors during the COVID-19 outbreak (ECLB-COVID19)” Electronic survey, with 1,047 responses reflecting an increase in sitting time from 5 to 8 h per day due to home isolation, and this article also had the highest annual citations.

Nearly 1/3 of the 50 most cited articles were from the United States, which can be attributed to (1) the tendency of researchers in the United States to prioritize citing their national sources when publishing articles (76), and (2) the fact that the United States is the country with the highest number of confirmed novel coronavirus pneumonia since the 2019 outbreak (77) and has invested abundant scientific funding. Unfortunately, COVID-19 broke out in Wuhan, China, but there are only two highly cited articles from China, including the Taiwan region.

An analysis of the co-authors of the top-50 cited articles showed that the authors who contributed to the study of COVID-19 and physical activity were mainly the team of Smith L, Musumeci G, and Napoli C. Their findings focused on (1) the association between changes in physical activity and mental status before and after the COVID-19 outbreak (35, 61, 64); (2) the effects of the COVID-19 pandemic on physical activity in different populations (28, 69); and (3) changes in physical activity induced by COVID-19 in college students (57, 68), which provided a great reference value for the subsequent studies in this field.

This study found that most articles were published in high-impact (≥3) journals, such as the *British Journal of Sports Medicine* and the *International Journal of Behavioral Nutrition and Physical Activity*, a sports medicine journal. Notably, the top-50 cited articles were published in a wide variety of journals, including public health (e.g., *International Journal of Environmental Research and Public Health*), psychology (e.g., *General Hospital Psychiatry*), nutrition (e.g., *American Journal of Clinical Nutrition*), and neurology (e.g., *Brain Behavior and Immunity*), and general journals (e.g., *Scientific Reports, PLoS One*), indicating that research on physical activity during the COVID-19 pandemic is a typically multidisciplinary intersection.

We also found that most studies focused on epidemic-induced changes in physical activity in different populations, the effect of physical activity on mental status, and the association between poor lifestyle habits and COVID-19. Understanding the different research areas of the top-50 cited articles is crucial, as it is crucial not only for journal editors to select and judge future scientific work but also for young researchers to publish effectively. Surprisingly, almost all the studies on the list were investigative studies, with fewer intervention studies addressing physical activity promotion during the COVID-19 pandemic. This may be related to the focus of earlier researchers on exploring potential factors for changing lifestyle habits. Considering the benefits of physical activity for the COVID-19 and general populations, the future trend will gradually shift to high-quality randomized controlled trials. Recently published guidelines provided general recommendations on physical activity, focusing on exercise training guidelines for isolated homes due to COVID-19 (78–80).

We performed a co-occurrence analysis of keywords in the top-50 cited articles by VOSviewer to describe the research hotspots based on the citation analysis. The network mapping showed that the focus around COVID-19 and physical activity was on (1) the lifestyle changes caused by the epidemic remained a hotspot; (2) the impact of the epidemic on residents' mental health (including depressive, anxiety, loneliness), especially the longitudinal trends, remained a direction to be explored; (3) the physical activity levels of different populations in the COVID-19 context, mainly for youth and children, but also cannot ignore the elderly and chronic disease population; (4) reliability and validity of different instruments in measuring physical activity changes, and assessment of physical activity by more accurate and objective accelerometers may be the future trend; (5) exercise-based physical activity for post-COVID, As Jimeno-Almazán et al. stated, ”*Exercise has been shown to be beneficial in multiple pathologies with which the post-COVID-19 syndrome shares similarities both in terms of symptoms and its possible pathogenic mechanisms, it is worth considering the potential favorable effect that this would bring in the recovery of these patients*“ (81).

This study has implications for public health. First, reducing exercise and other physical activity due to the blockade policy of the outbreak may have potentially adverse effects on the physical and mental health of children and adolescents, and the resumption of physical activity by young people should be promoted after COVID-19 (82). Second, a sedentary lifestyle is potentially a significant cause of cardiovascular disease, the leading cause of death. The outbreak of COVID-19 may worsen this situation because isolation decreases habitual and recreational physical activity. Therefore, Crisafulli and Pagliaro suggest that jogging alone (wearing a mask) could gradually increase when the epidemic improves (i.e., when the prevalence and increase of new cases are significantly reduced) (83). Third, frailty is a common condition in the elderly and chronically ill populations, and physical inactivity can exacerbate the worsening of frailty (84). In the dual context of the epidemic and healthy aging, policymakers should pay more attention to the physical activity management in these two vulnerable groups.

The present study has a strength in that, based on 50 classical citations, it is more worthwhile for researchers to analyze the hot spots of research on COVID-19 and physical activity. Inevitably, there are also some limitations. First, the databases in our study were limited to the Web of Science Core Collection, resulting in the absence of other “classic” articles. Second, we did not analyze inter-institutional collaborations, which may have reduced the contribution. The third limitation is the inherent bias of citation analysis. The total citations may increase over time, which means that older publications will undoubtedly receive more citations than newer ones (85).

## Conclusion

This study analyzed the 50 most cited articles on COVID-19 and physical activity through bibliometric citation analysis. The results provide a landscape of physical activity research in global outbreaks of novel coronaviruses and identify substantial progress.

## Data availability statement

The original contributions presented in the study are included in the article/supplementary material, further inquiries can be directed to the corresponding author/s.

## Author contributions

FZ and WL: conception and design. YY and YZ: collection data. FZ: manuscript writing. WL and HZ: manuscript revise. All authors had read and approved the final manuscript and agreed on its submission.

## Funding

This study was supported by the Longhua Hospital Shanghai University of Traditional Chinese Medicine (Grant No. Y21026) and Shanghai University of Traditional Chinese Medicine (Grant No. 2022YJ-10).

## Conflict of interest

The authors declare that the research was conducted in the absence of any commercial or financial relationships that could be construed as a potential conflict of interest.

## Publisher's note

All claims expressed in this article are solely those of the authors and do not necessarily represent those of their affiliated organizations, or those of the publisher, the editors and the reviewers. Any product that may be evaluated in this article, or claim that may be made by its manufacturer, is not guaranteed or endorsed by the publisher.

## Abbreviations

COVID-19: 2019 novel coronavirus.
